# Rational Drug Design and Synthesis of Molecules Targeting the Angiotensin II Type 1 and Type 2 Receptors

**DOI:** 10.3390/molecules20033868

**Published:** 2015-03-02

**Authors:** Tahsin F. Kellici, Andreas G. Tzakos, Thomas Mavromoustakos

**Affiliations:** 1 Department of Chemistry, National and Kapodistrian University of Athens, Panepistimiopolis Zografou 15771, Greece; 2 Department of Chemistry, University of Ioannina, Ioannina 45110, Greece

**Keywords:** angiotensin II receptors, AT_1_R, AT_2_R, rational drug design, synthesis, molecular modeling, drug-membrane interactions

## Abstract

The angiotensin II (Ang II) type 1 and type 2 receptors (AT_1_R and AT_2_R) orchestrate an array of biological processes that regulate human health. Aberrant function of these receptors triggers pathophysiological responses that can ultimately lead to death. Therefore, it is important to design and synthesize compounds that affect beneficially these two receptors. Cardiovascular disease, which is attributed to the overactivation of the vasoactive peptide hormone Αng II, can now be treated with commercial AT_1_R antagonists. Herein, recent achievements in rational drug design and synthesis of molecules acting on the two AT receptors are reviewed. Quantitative structure activity relationships (QSAR) and molecular modeling on the two receptors aim to assist the search for new active compounds. As AT_1_R and AT_2_R are GPCRs and drug action is localized in the transmembrane region the role of membrane bilayers is exploited. The future perspectives in this field are outlined. Tremendous progress in the field is expected if the two receptors are crystallized, as this will assist the structure based screening of the chemical space and lead to new potent therapeutic agents in cardiovascular and other diseases.

## 1. Introduction

**Figure 1:**
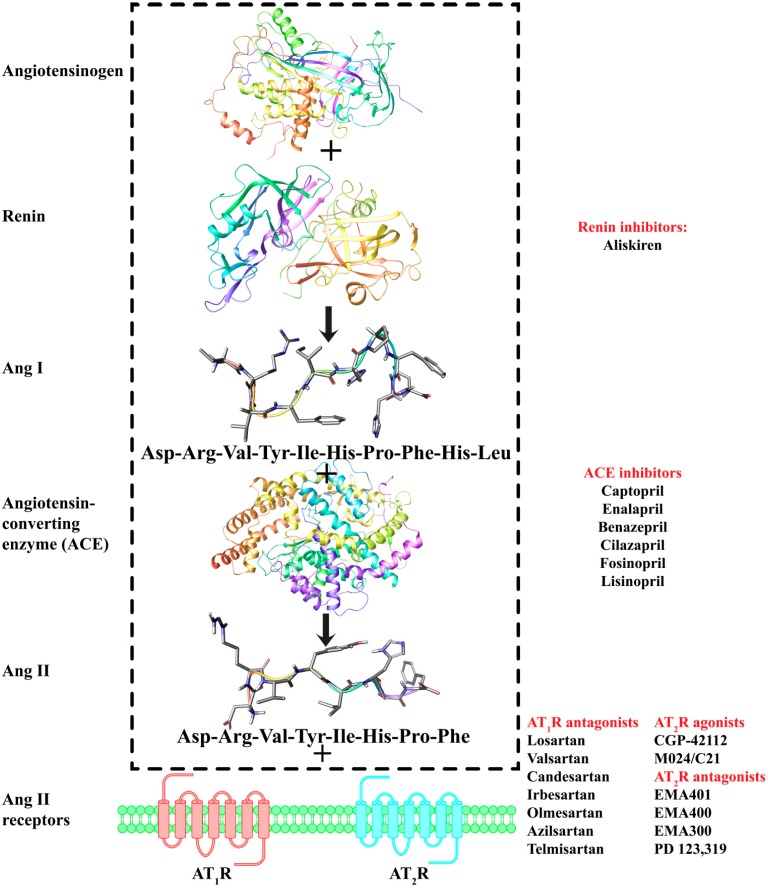
The RAS and representative drugs that target this system.

The renin angiotensin system (RAS) is the main target for regulating the body’s blood pressure [1]. This is a bioenzymatic system where angiotensinogen, through the two enzymes renin and angiotensin converting enzyme (ACE), is converted to the octapeptide hormone angiotensin II (Ang II) that in pathological conditions causes vasoconstriction. ACE inhibitors were the first drugs designed and synthesized to block the detrimental effects of Ang II (a compound word of Greek origin composed of the words «αγγείο» (vessel) and «τάση» (tension)) (Figure 1) [2]. 

Recently, the efforts to design and synthesize renin inhibitors were met with success with the discovery of the drug aliskiren [3]. Today, we have gained more knowledge on RAS: First, we know that more biochemical pathways are affecting the conversion of angiotensinogen to Ang II; second, although Ang II affects mainly two GPCR receptor subtypes, namely AT_1_R and AT_2_R, at least four different subtypes have been identified (designated as AT_1_R, AT_2_R, AT_3_R and AT_4_R [4]). Also, the different metabolites of Ang II, which form after proteolytic degradation of the parent molecule, present biological activity. In addition, Ang II has high binding affinity to neurolysin which in turn may affect significantly the activity on RAS [5,6,7]. The action of Ang II on AT_1_R was the first to be studied in detail, while the mode of action of AT_2_R remained elusive for a long time owing to the lack of ligands that selectively target this receptor as also due to its low expression [8,9,10,11]. The recent discovery of novel ligands that selectively target these receptor subtypes has paved the way to deconvolute the functions of AT_1_R and AT_2_R. Furthermore, new functions of the two receptors have been revealed. It is now shown that AT_1_R and AT_2_R present opposing biological functions, e.g., AT_2_R has anti-proliferative properties [12,13], while AT_1_R facilitates angiogenesis and cellular proliferation [12,14]. Furthermore, besides the classical functions mediated by the AT_1_R like vasoconstriction, proliferation of vascular smooth muscle and cardiac cellular growth, a direct correlation has been identified between the up-regulation of AT_1_R and the immunosuppression and invasiveness state in many cancer types, establishing AT_1_R as a potential cancer drug target [15,16,17,18,19]. Respectively, the discovery of new ligands that selectively target AT_2_R has encouraged the scientific community to explore in detail functions orchestrated and associated by AT_2_R. For instance, AT_2_R adopts a protective role in pathological conditions such as tissue injury and inflammation [20], diabetic neuropathy [21], stroke damage [22], diabetes type 2 [23], spinal cord injury [24] and cancer [25,26]. The accumulating body of evidence highlights the enhanced potential of both AT_1_R and AT_2_R to act as important pharmaceutical targets for a diverse array of pathologies. In light of this, it is worth collating the principles that govern selectivity on these two subtype receptors and utilize these to design and develop the new generation of more selective compounds.

**Figure 2:**
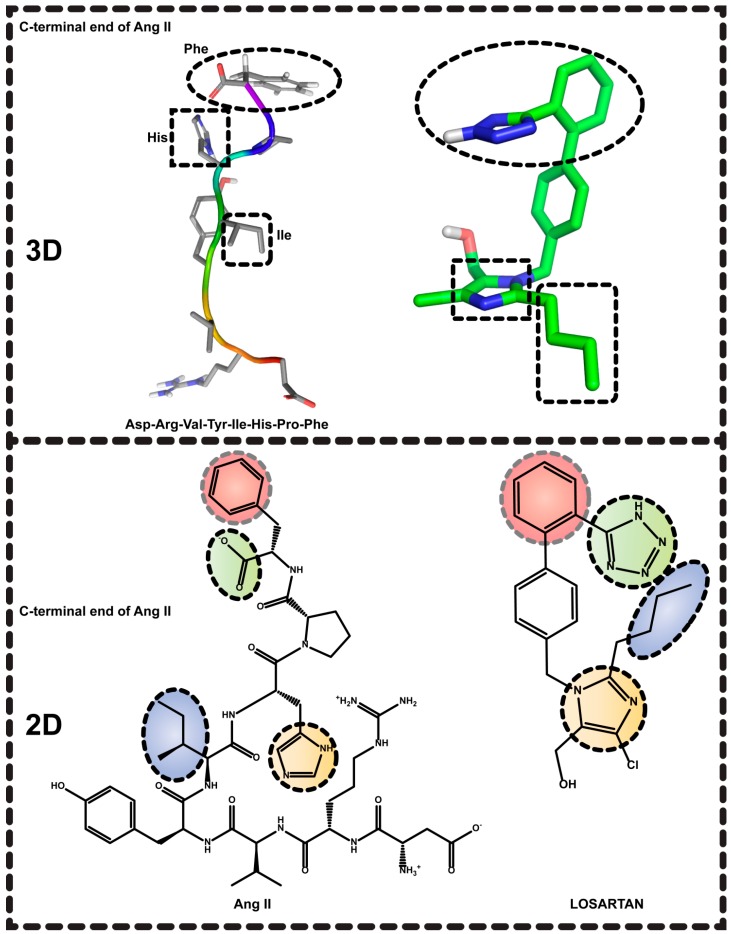
(top, left) A 3D proposed model of Ang II. (top, right) A 3D structure of losartan. Circled or squared segments of the two molecules illustrate common pharmacophores. Circled common pharmacophores are shown for the 2D structures of Ang II (top, left) and losartan (bottom, right).

As with renin and ACE inhibitors, extensive rational design plans had to be implemented by researchers working both in industry and academia to discover AT_1_R antagonists. Initially, efforts were mainly focused on peptides, but owing to the known disadvantages that peptides encounter they could not enter clinical trials or the market as drugs. However, these studies provided valuable SAR knowledge. From peptides, the scientists have been led to small organic molecules that mimicked the C-terminal segment of Ang II (Figure 2).

**Figure 3:**
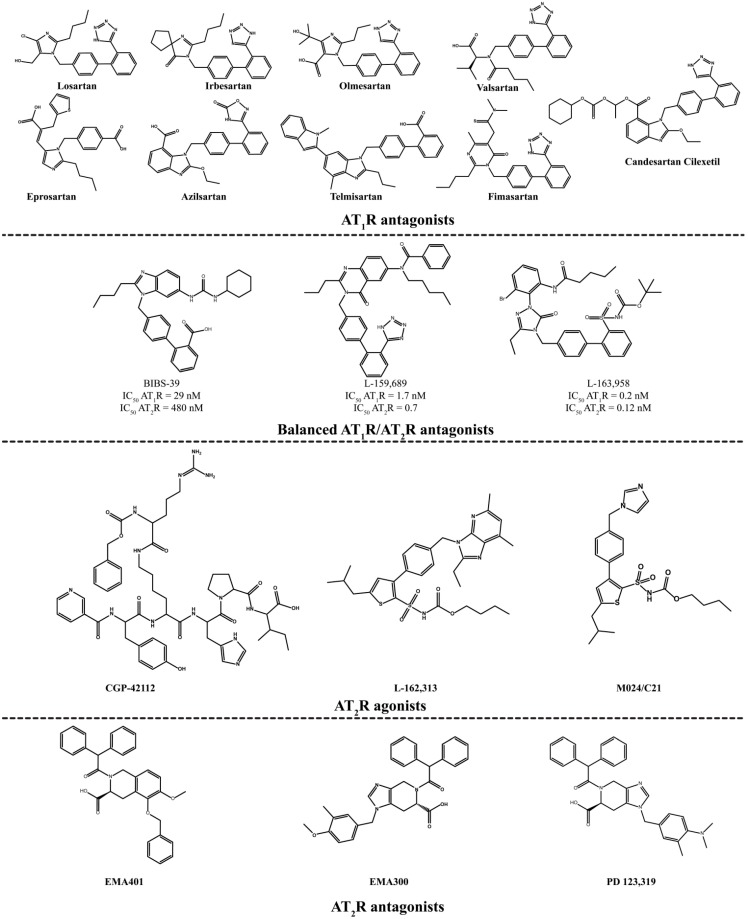
AT_1_R antagonists, AT_1_R/AT_2_R hybrids, AT_2_R antagonists and agonists.

The first AT_1_R antagonist that entered the market was losartan [IC_50_ = 5.5 nM [27] (binding to human AT_1_R using as radioligand ^125^I[Sar^1^-Ile^8^]Ang II), 150 nM [28] (inhibition of [^125^I]Ang II (0.2 nM) binding to bovine adrenal cortical membranes), 6.7 nM [29] (displacement of [^125^I]Sar^1^,Ile^8^-Ang II specifically bound to AT_1_R in rat hepatic membranes)]. The success of losartan followed eight more derivatives constituting the class of SARTANs or angiotensin receptor blockers (ARBs) (Figure 2 and Figure 3).

As mentioned above, for a long time the scientific community neglected AT_2_R and its major physiological role remained elusive [9,30,31,32]. However, the design and synthesis of the selective AT_2_R antagonist PD 123,319 [33] and the selective AT_2_R agonist CGP-42112A [34], the selective agonist M024/C21 [35] and the selective AT_2_R antagonist EMA401 that entered clinical trials for the treatment of neurophathic pain [36] led to an understanding of the physiological role of this receptor and the design and synthesis of molecules possessing beneficial effects [36,37]. Ichiki *et al.* and Rein *et al.* [38,39] reported that mice lacking AT_2_R exhibited a higher BP and higher sensitivity to an angiotensin II-induced hypertensive effect than normal mice. This information triggered the effort towards the discovery of AT_2_R agonists as antihypertensive targets. Speth *et al.* [40] synthesized [*p*-amino-Phe6] Ang II and more recently Yamada *et al.* [41], discovered the peptide novokinin as an AT_2_R agonist possessing antiopioid and anorexigenic activity only on AT_2_R.

Novokinin (RPLKPW) was designed based on ovokinin (FRADHPFL), a vasorelaxing peptide derived from ovalbumin. Its vasorelaxing activities were blocked by PD123,319, an AT_2_R antagonist. Guimond *et al.* [42], recently proved that the octapeptides saralasin and sarile act as AT_2_R agonists. A method to regulate ligand selectivity for AT_1_R and AT_2_R through controlling aromatic–prolyl interactions in Ang II, by tuning aromatic electronics has recently been presented [26]. This method was used to investigate substituent effects on the *para*-position of phenylalanine installed at position 6 of Ang II (4-x-Phe^6^). The analogue bearing an electron donor (–OH) group was a highly selective AT_2_R agonist with a *K*_i_ of 3 nM that also pinpointed inhibitory activity against breast carcinoma cell proliferation at nM concentration.

Overproduction of Ang II in pathological states has harmful effects on many systems, and the design and synthesis of more selective AT_1_R antagonists and AT_2_R agonists and antagonists is expected to lead to drugs with multiple benefits. Already, AT_1_R antagonists in the market are clearly characterized by their antihypertensive effects [43].

This review article focuses on recent efforts on the design and synthesis of small bioactive molecules that can act on AT_1_R and AT_2_R. For molecules synthesized before 2010, which act on AT_1_R, the reader can get detailed information from the cited review [44]. Emphasis will be also given to recent synthetic efforts to develop multitarget drugs that have structural components recognized by AT_1_R and AT_2_R [45].

In addition to the synthetic efforts in the recent years, parallel attempts have been made to model the two receptors using new approaches. Without doubt, these approaches can aid the design and synthesis of new molecules acting on the two receptors. Review articles covering these efforts until 2011 can be found in the literature [44,46], and thus this review will focus on the efforts after 2011.

Finally, general conclusions on the future prospects of the recent discoveries will be presented. Our hope is that this article will assist medicinal chemists to design, synthesize and discover new drugs possessing beneficial effects for major diseases such as cardiovascular and cancer.

## 2. Results and Discussion

### 2.1. Novel Synthetic Molecules Acting on the AT_1_R

**Scheme 1:**
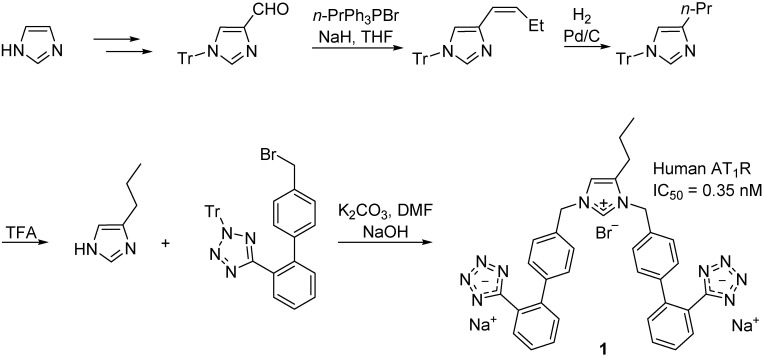
Outline of the synthesis of the imidazolium **1** (named BV6 in the published article [47]). Experiments were performed with the human AT_1_R and the radioligand ^125^I[Sar^1^-Ile^8^]Ang II (1000 nM).

**Figure 4:**
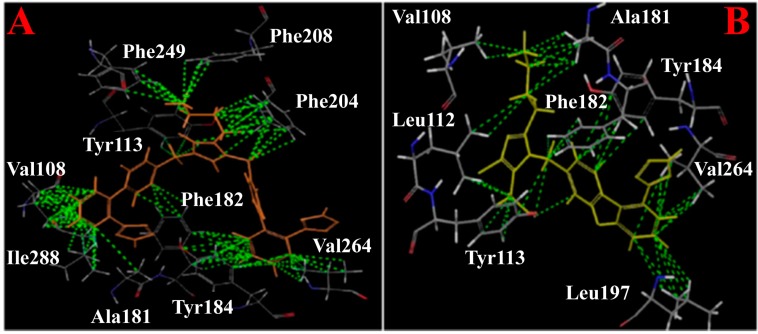
The hydrophobic interactions of the imidazolium **1** in **A** and of losartan in **B** with AT_1_R. Imidazolium **1** exerts more hydrophobic interactions owing to its additional biphenyl tetrazole segment.

Agelis *et al.* [47,48] synthesized a series of symmetrically bis-substituted imidazole analogues bearing at N-1 and N-3 two biphenyl moieties *ortho*-substituted either with tetrazole or carboxylate groups. Among them, the imidazolium **1** (BV6, Scheme 1) showed superior antagonistic activity and receptor affinity to that of losartan [47,49]. The same team studied the interactions of the imidazolium **1** with lipid bilayers and its delivery into a mesoporous silicate drug delivery matrix SBA-15 [49]. Both its interactions with membranes and delivery properties point out that its structural modifications may lead to a drug molecule. The imidazolium **1** was accommodated in AT_1_R with more favored lipophilic interactions with respect to the prototype losartan which may account for its high activity (Figure 4).

**Scheme 2:**
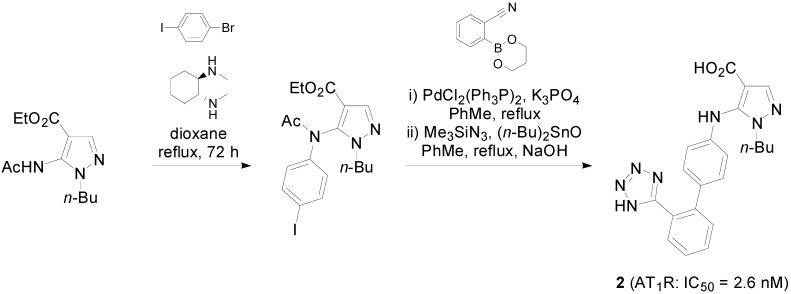
Outline of the synthesis of **2**. The binding activity of the compound was determined in human recombinant AT_1_R using as radioligand ^125^I[Sar^1^,Ile^8^] Ang II (0.05 nM).

The synthetic molecule **2** (Scheme 2) bears a bioisosteric replacement of the CH_2_ bridge between the biphenyl system and the nitrogen heterocyle by an NH moiety [50].

**Scheme 3:**
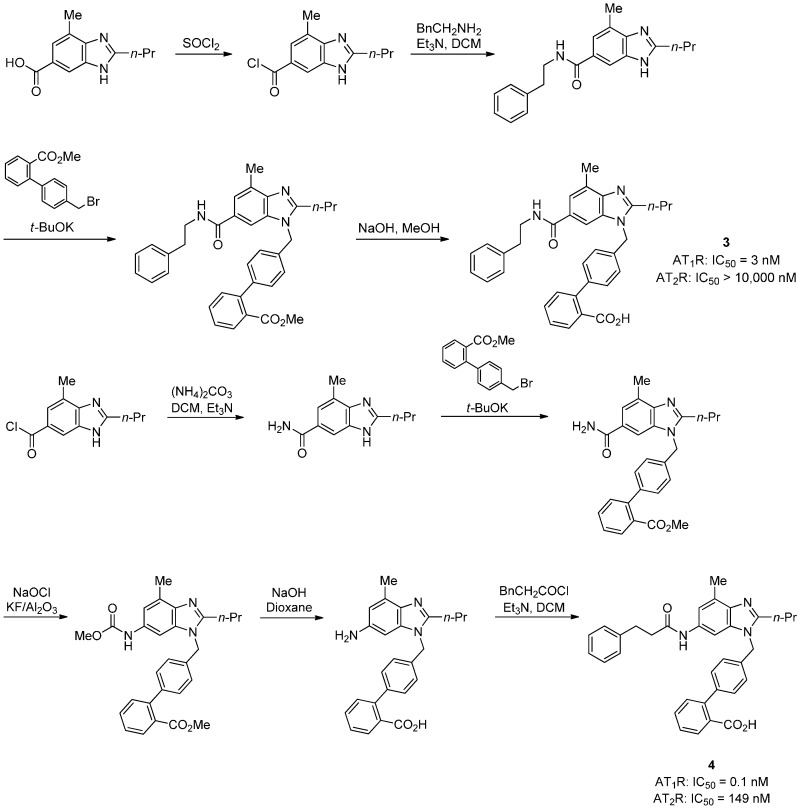
Outline of the synthesis of of **3** and **4**. The binding activities of the compounds were determined in AT_1_R and AT_2_R using as radioligand ^125^I[Sar^1^,Ile^8^] Ang II (25 pM).

Compounds **3** and **4** (Scheme 3) were synthesized by Zhang *et al.* [51], and are promising selective AT_1_R antagonists. Da *et al.* [52], synthesized fluorine substituted derivatives of losartan, valsartan and irbesartan with carboxylic acid group as replacement to the known potent tetrazole moiety at the 2'-biphenyl position. 

**Scheme 4:**
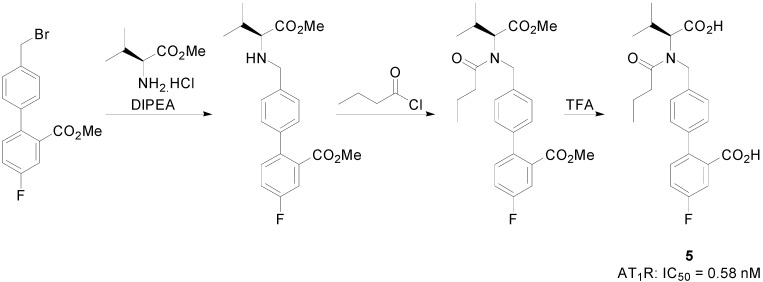
Outline of the synthesis of the biphenyl **5**. Binding assays were performed using vascular smooth muscle cells obtained from thoracic aorta of rats, using ^125^I[Sar^1^,Ile^8^] Ang II as radioligand at a concentration of 0.1 nM.

The biphenyl **5** (Scheme 4) showed an efficient and long lasting effect in reducing blood pressure which lasted more than 24 h at a dose of 10 mg/kg in spontaneous hypertensive rats (SHR), which was much better than controls losartan and valsartan. In addition to antihypertensive property, the biphenyl **5** also inhibited prostate cancer *in vitro* and *in vivo*.

**Scheme 5:**
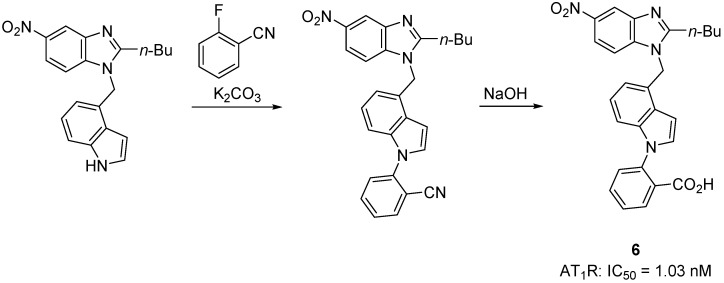
Outline of the synthesis of the benzimidazole **6**. The radioligand binding assay were performed using vascular smooth muscle cells obtained from thoracic aorta of rats, using ^125^I[Sar^1^,Ile^8^] Ang II (0.1 nM) as radioligand.

The 5-nitrobenzimidazole **6** (Scheme 5) exerts high nanomolar and durable activity (IC_50_ = 1.03 ± 0.26 nM) in vascular smooth muscle cells [53]. This compound bears an indole benzoic ring instead of the biphenyl scaffold with an acidic segment attached at the *ortho* position, (a common feature to commercial drugs except eprosartan that contains only one phenyl ring) [53].

**Scheme 6:**
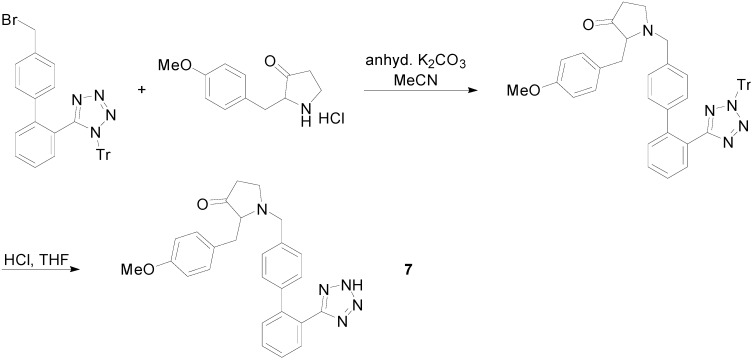
Outline of the synthesis of the pyrrolidone **7**

New AT_1_R antagonists were designed and evaluated based on a central pyrrolidine system bearing biphenyl-tetrazoles or biphenylcarboxylic acids at the N12, C-3 and C-4 positions [54]. Among them compound **7** (Scheme 6) was the most promising and had 2-fold higher hypotensive activity than losartan and similar level of antihypertensive activity to losartan with LD_50_ value of 117 μg/Kg demonstrating in this way the high safety margin of the compound. The compound was evaluated *in vivo* for hypotensive activity on normotensive rats.

**Scheme 7:**
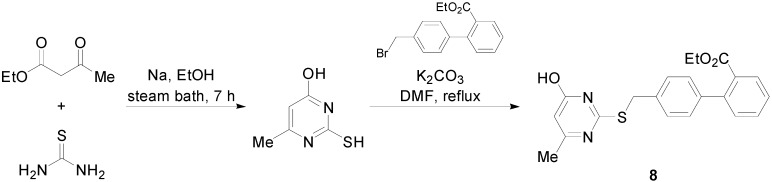
Outline of the synthesis of compound **8**.

A series of compounds based on the α1-adrenoreceptor antagonist drug urapidil and molecular modeling were synthesized. Compound **8** (Scheme 7) exhibited hypotensive activity more or less similar to losartan [55]. The *in vivo* biological evaluation was carried out on normotensive adult rats.

The presented examples illustrate that there is still space for developing novel bioactive AT_1_R antagonists. Novel ideas such as the design and synthesis of molecules bearing more lipophilic segments that fit in the AT_1_R certainly will provide impetus in the field.

### 2.2. AT_2_R Agonists and Antagonists

Establishing ligands that will present enhanced selectivity for AT_2_R *vs.* AT_1_R is based on the fact that AT_2_R antagonizes the functions of AT_1_R. Activation of AT_2_R leads to apoptosis, antiproliferation and vasodilation, whereas activation of AT_1_R leads to cellular growth, proliferation and vasoconstriction [26].

These results have highlighted the potential of AT_2_R to act as a novel drug target in tissue regeneration and protection through controlling apoptosis, fibrosis and inflammation [56], heart failure [57], and cancer [25,26].

**Figure 5:**
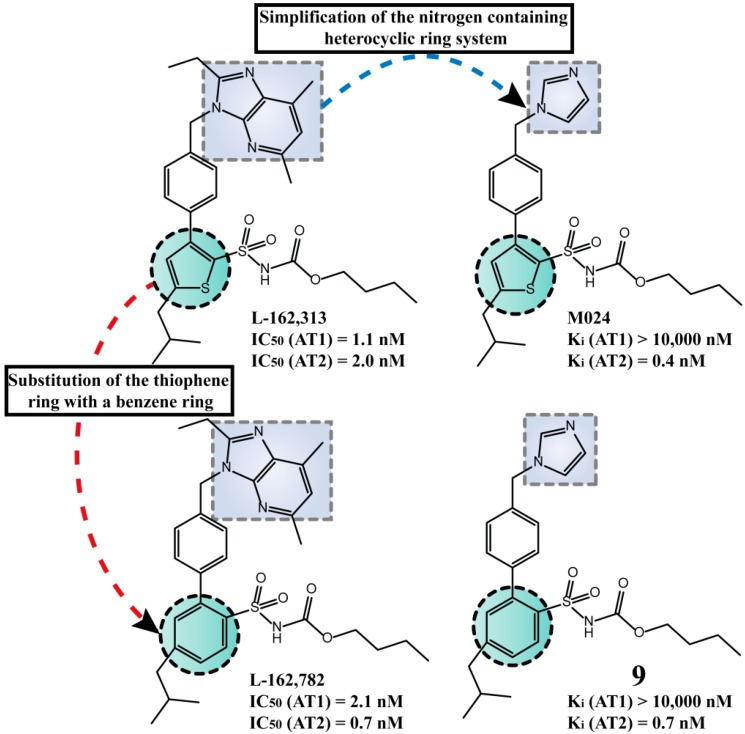
Structural modifications performed on the original scaffold to lead to the highly selective AT_2_R agonist **9**. The activity of compound **9** was evaluated in binding assay using AT_1_R from rat liver membranes and AT_2_R from pig uterus membranes, while [^125^I]Ang II (0.03 nM) was used as radioligand.

**Scheme 8:**
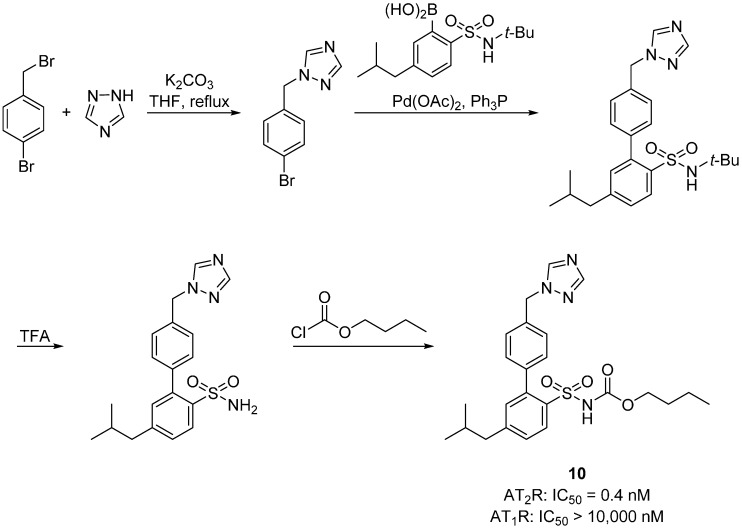
Outline of the synthesis of **10**. AT_1_R affinity assay was determined using rat liver membrane, while porcine myometrial membrane was used for AT_2_R. [^125^I]Ang II was used as radioligand (0.03 nM).

Wan *et al.* synthesized the first selective nonpeptide AT_2_R agonist M024/C21 by stepwise simplification of the nitrogen containing heterocyclic ring system [11,35,58,59]. The substitution of the thienyl-phenyl to the biphenyl scaffold (resembling L-162,782, Figure 5) produced the equipotent compound **9** [*K*_i_ (AT_1_R) > 10,000 nM, *K*_i_ (AT_2_R) = 0.7 nM] [58,60] showing that the two scaffolds are bioisosteric in these compounds. Compound **10**, a derivative of L-162,782, was synthesized by Liu *et al.* [61], in an attempt to develop new AT_2_R agonists as novel antihypertensive candidates (Scheme 8). The compound was superior to the reference drug losartan in SHRs and it had no significant impact in heart rate.

**Scheme 9:**
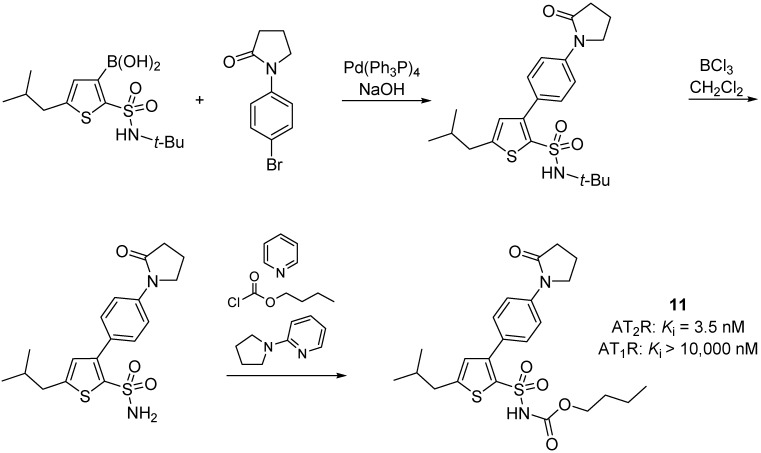
Outline of the synthesis of compound **11**. Affinity assay was conducted as descripted in the previous example.

Mahalingam *et al.* [62], synthesized derivatives of AT_2_R agonist M024/C21 (Figure 3) in an attempt to reduce the CYP450 inhibitory property. The best analogue prepared was compound **11** (Scheme 9) which induced neurite elongation in NG 108-15 cells and served as a potent and selective AT_2_R agonist.

**Figure 6:**
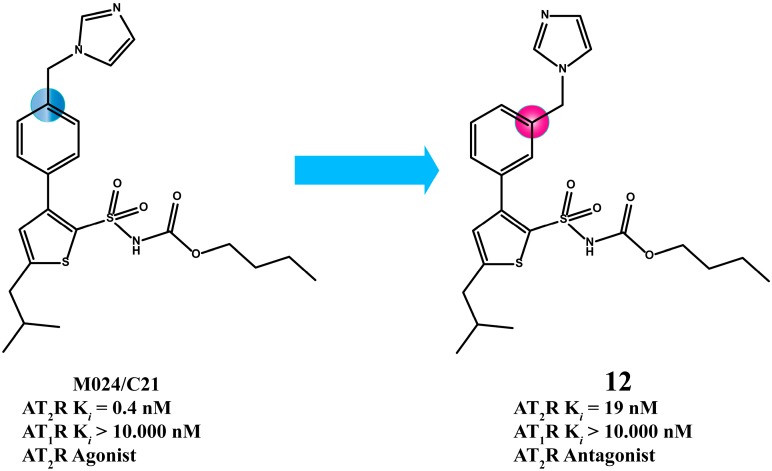
The analogue **12** that bears a *meta*-substituted phenyl is an AT_2_R antagonist, in contrast to M024/C21 which bears *para*-substituted phenyl and is an AT_2_R selective agonist. The dissociation constant *K*_i_ values were determined as described in the previous example.

Murugaiah *et al.* synthesized novel compounds derivatives of M024/C21 which are *meta*- rather than *para*-substituted on the phenyl [35,63,64]. Interestingly, they generated AT_2_R antagonists in comparison to M024/C21, which is a selective AT_2_R agonist. Compound **12** is a selective AT_2_R antagonist (Figure 6).

**Figure 7:**
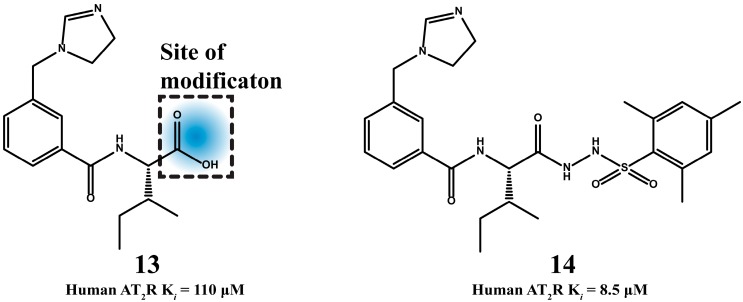
The synthetic compound **14** possesses improved AT_2_R activity compared to the lead compound **13**.

Veron *et al.* [65], used compound **13** as a lead to synthesize sixteen new C-terminally modified analogues. Compound **13** bears structural similarities with the C-terminal segment of Ang II. Specifically, it contains a carboxylate group as Phe^8^, isoleucine side chain instead of benzene of Phe^8^ and imidazole ring as His^6^ [66]. Compound **14** proved the most active and was over 12-fold more potent than the lead compound **13** (Figure 7). All the synthesized compounds were evaluated for their human AT_2_R affinity in a radioligand binding assay measuring the displacement of [^125^I]CGP-42112A, a selective AT_2_R agonist.

**Scheme 10:**
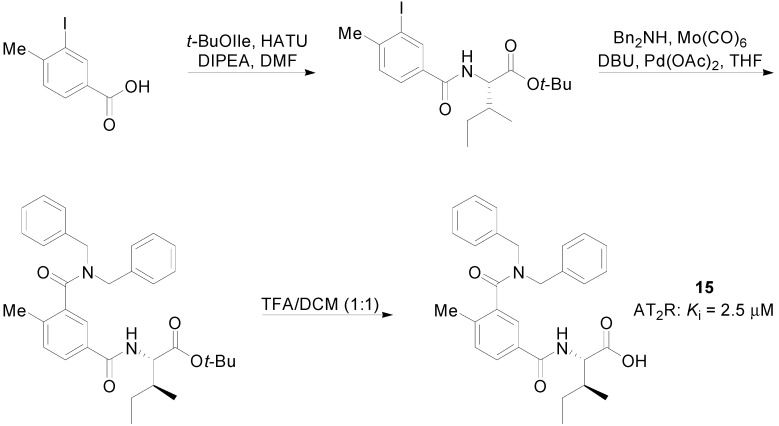
Outline of the synthesis of compound **15**.

Behrends *et al.* [67], also used compound **13** as a lead to evaluate fifteen new synthetic compounds. Thirteen of them showed higher activity than the lead compound **13**. An example of the synthesis of analogue **15** is given below (Scheme 10). Binding assays were performed with recombinant human AT_2_R or AT_1_R and relying on the displacement of [^125^I]CGP-42112 (AT_2_R) and [^125^I][Sar^1^, Ile^8^] Ang II.

From the above examples, it is clear that the discovery of new AT_2_R agonists and antagonists is still in its infancy. Only recently the interest was increased on AT_2_R and of course more time is needed to tune the requirements for selectivity of AT_2_R agonism/antagonism *vs.* AT_1_R agonism/antagonism.

### 2.3. Multitarget Drugs

As cardiovascular diseases involve complex pathways, polypharmacology approaches can be a fruitful direction to tackle them [68]. Already, numerous multitarget drug molecules, following the molecular hybridization approach combining two discrete drugs in one molecule, have been designed and synthesized with beneficial effects. Some such examples are outlined below.

Mojarrad *et al.* [69], designed and synthesized molecules that combine structural elements present in AT_1_R antagonist and 1,4-dihydropyridine calcium channel blockers. Among the synthesized molecules, eight showed both calcium channel and AT_1_R blocking activities. 

**Figure 8:**
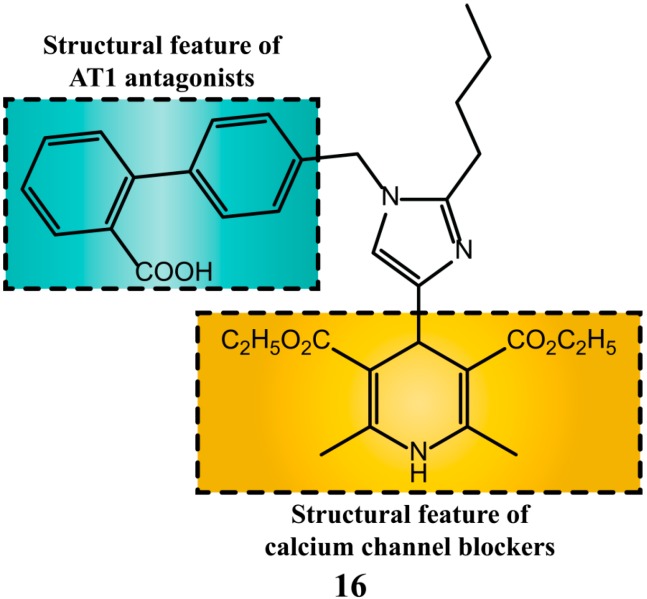
Compound **16** was designed with structural features to act as AT_1_R antagonist and calcium channel blocker.

Interestingly, the effects of analogue **16** on AT_1_R were 100,000 higher than losartan (Figure 8). The experiments were conducted using aortic rings contracted by Ang II. The relaxant effects were measured and compound **16** had pD2 value 100,000 times higher (losartan pD2 = −5.66 ± 0.16 and 16 − 10.52 ± 0.18) [70].

**Figure 9:**
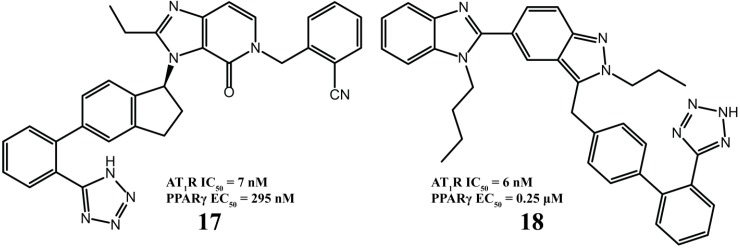
Molecules designed to act as AT_1_R antagonists and partial PPAR-γ agonists. Activation of human PPARγ was evaluated using a chimeric receptor PPARγ Ligand Binding Domain (LBD)/Gal4 DNA Binding Domain while the activation of human recombinant AT_1_R was determined in a competition radioligand binding assay with [^125^I]Tyr^4^-Sar^1^,Ile^8^-Ang II.

Compounds **17** and **18** (Figure 9) exert potent dual activity, AT_1_R antagonism and partial proliferator-activated receptor-γ (PPARγ) agonism and have desirable ADME properties [71,72].

**Scheme 11:**
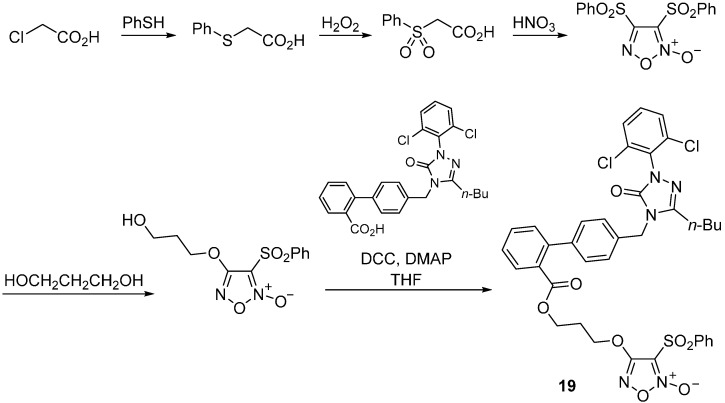
Outline of the synthesis of compound **19**.

A series of nitric oxide donating derivatives of [1,2,4] triazol-5(4*H*)-one exert both high AT_1_R antagonist activity and good maximum NO release [73]. The synthesis of the most promising compound **19** is outlined in Scheme 11.

A very interesting chapter has been written by Murugesan describing the design of novel dual angiotensin II and endothelin receptor type A (ETA) antagonists [74]. Since both Ang II and endothelin I increase the blood pressure (the first acting in the AT_1_R and the other on the ETA) a blockade of both pathways could lead to a more effective reduction of blood pressure.

Multitarget drugs will certainly continue to be an interesting and fruitful approach and potentially can lead to more beneficial drugs with fewer side effects. At the moment only the structural requirements for AT_1_R antagonism are utilized. A deeper knowledge on the molecular determinants on the AT_2_R agonism and antagonism in the future will offer to medicinal chemists enhanced versatility towards the design and synthesis of new generation of more potent compounds.

### 2.4. Molecular Modeling

Ohmo *et al.* [75] demonstrated a unique binding mode of telmisartan to AT_1_R. In particular, it was shown that telmisartan firmly binds to the AT_1_R through a unique shape that resembles the Greek letter delta (Δ) and it locks favorably at the receptor (delta lock), which may explain its superiority to other ARBs in halting cardiovascular diseases in patients with hypertension. The AT_1_R was modeled using the bovine rhodopsin crystal structure as a template and the docking program GOLD.

Kritsi *et al.* [76] using NMR spectroscopy, molecular modeling and Differential Scanning Calorimetry) (DSC) illustrated that telmisartan adopted a unique fingerprint in terms of its receptor interactions combined with the bilayer effects in comparison to other AT_1_R antagonists.

**Figure 10:**
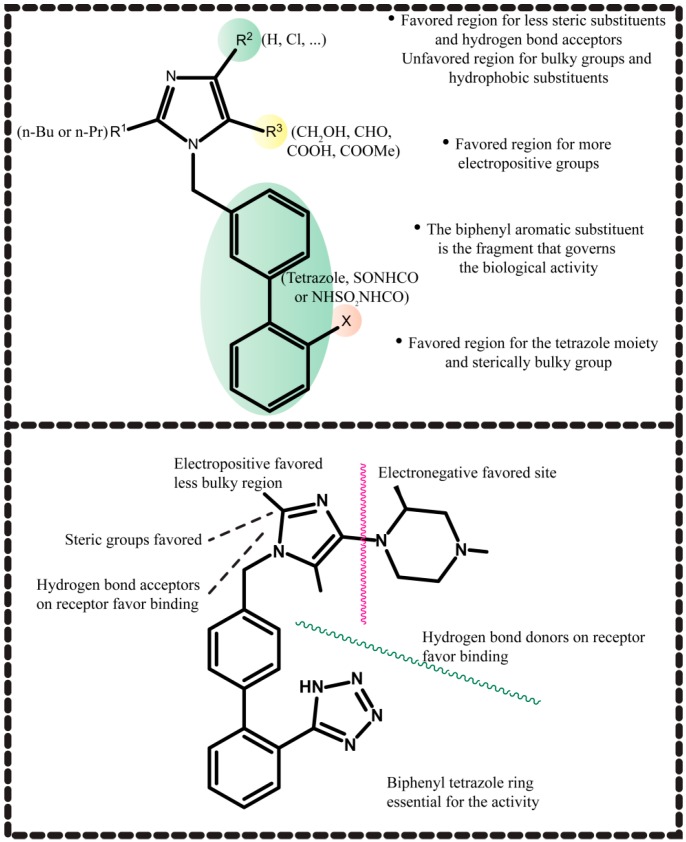
Requirements for AT_1_R antagonism as are depicted by the series of compounds used by Sharma *et al.* using descriptor optimization, PLSR and KNN-HFA analysis as well as 2D G-QSAR and 3D QSAR studies.

Stepwise and simulated annealing were applied for descriptor optimization while PLSR and KNN-HFA analysis were used for 2D, G-QSAR and 3D-QSAR model development on acyl sulfonamides and acyl sulfamides derivatives of imidazole [77]. The major conclusions of this study are outlined in Figure 10. Sharma *et al.* [78,79,80] also published a series of articles in which QSAR models are constructed and evaluated.

**Figure 11:**
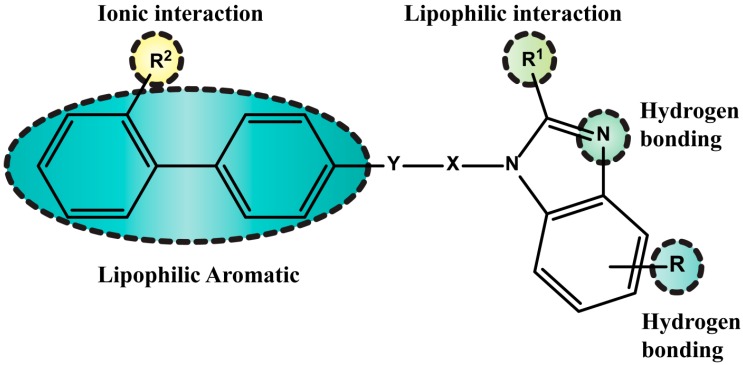
Important structural features for AT_1_R antagonism in the molecules studied by Vyas *et al.* using CoMFA and CoMSIA studies.

**Scheme 12:**
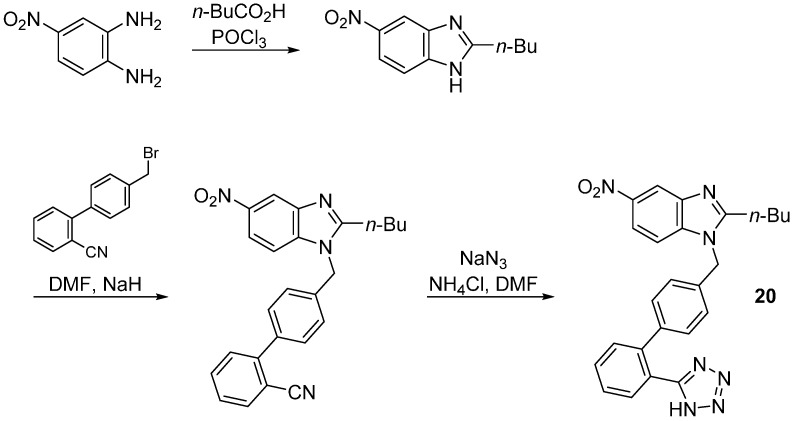
The steps involved in the synthesis of **20** using as starting material 4-nitrobenzene-1,2-diamine.

Vyas *et al.* [81] designed novel substituted benzimidazole derivatives that serve as AT_1_R antagonists based on CoMFA and CoMSIA studies and *in silico* pharmacokinetic properties and toxicities. The most promising features for drug activity of this series of compounds are shown in Figure 11. Among the molecules they designed, they synthesized the two most promising. The synthesis of the most potent of the two, which possessed effects comparable with telmisartan, is outlined in Scheme 12. Compound **20** was evaluated using isolated rat aortic ring to determine its activity to competitively inhibit Ang II binding to AT_1_R. The antagonistic activity, expressed as pA2 values, was 7.1 ± 0.31 for compound **20** and 7.5 ± 0.29 for telmisartan.

**Figure 12:**
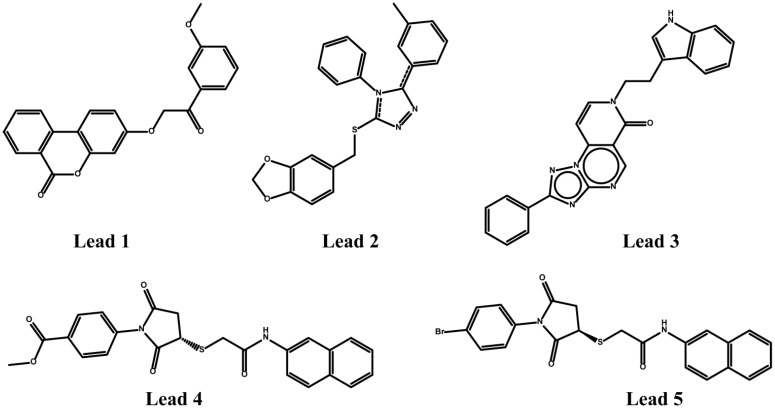
Five leads acting both at AT_1_R and ETA receptors using molecular modeling approach combining quantum-polarized ligand docking (QPLD), MM/GBSA free-energy calculation and 3D-QSAR analysis.

Singh *et al.* [82] applied molecular modeling approach combining quantum-polarized ligand docking (QPLD), MM/GBSA free-energy calculation and 3D-QSAR analysis to evaluate 24 compounds as dual AT_1_R and ETA antagonists and to reveal their binding modes and structural basis of the inhibitory activity. Pharmacophore-based virtual screening and docking studies led to five lead compounds (Figure 12) that are possible potent dual antagonists [82].

Silva *et al.* [83] applied docking and CoMFA studies on AT_1_R antagonists. The obtained model enables the design of new bioactive compounds with optimized pharmacological /biological properties.

Fimasartan, (Figure 3) a derivative of losartan (IC_50_ = 0.16 nM AT_1_R and selectivity index for AT_1_R over AT_2_R = 431,250) was approved for the treatment of hypertension by the Korean Food and Drug Administration in September 2010. 3D-QSAR studies were performed by Choi *et al.* [84] to guide the design of backups (derivatives of fimasartan that lack its side effects), in the case of unexpected side effects during phase 4 trials.

Sköld *et al.* [85] explored the binding modes of Ang II and some pseudopeptide analogues at AT_2_R and found two plausible binding modes in agreement with most of the suggested ligand-receptor contact points reported in the literature. Docking calculations, using MacroModel’s Monte Carlo multiple search minimum, showed that the ionic bridge between Phe^8^(carboxyl) of Ang II and Lys215 (AT_1_R) occurred most frequently in the ligand–receptor configurations. The contact between His^6^ (Ang II)-Asp279 (AT_1_R) and Arg^2^ (Ang II)-Asp297 (AT_1_R) and the contact of the Tyr^4^ residue of Ang II with Asp279 (AT_1_R) were among the most frequently observed.

Without doubt the pharmacophore approach, the use of molecular dynamics and calculation of thermodynamic parameters as well as 3D QSAR studies will lead to a better understanding of the structural requirements for the two Ang II receptors. At the moment the 3D QSAR studies are restrained mainly to AT_1_R and AT_2_R. However, this approach has to be extended to multitargeting since beneficial molecules have already been synthesized.

### 2.5. Mechanisms of Activation of AT_1_R and AT_2_R and Signalling

A recent review by Saku *et al.* [86] discusses the progress in the understanding of the molecular mechanism of AT_1_R and AT_2_R. The new scientific evidence according to the authors may lead to more selective therapeutic agents *i.e.*, inhibit AT_1_R function such as cell signaling, inhibit homo-dimerization, or overexpress AT_2_R-induced AT_1_R antagonism. A recent review by Akazama *et al.* [87] describes the progress in the receptor activation and signaling.

Matsoukas *et al.* [88,89] performed MD simulations with the AT_1_R—Ang II—lipids complex and observed a series of dynamic changes in the topology of Ang II and the intracellular part of the receptor. Complete binding profiles of Ang II to the AT_1_R, as well as the key transitional elements of the receptor and the agonist peptide upon activation through NMR and *in silico* studies were revealed. In addition, the use of a combination of computer simulations, including pharmacophore modeling, docking, molecular dynamics simulations with explicit inclusion of the membrane and ligand binding free energy calculations, permitted to propose the molecular mechanisms by which sartans interact with AT_1_R [89]. Sokkar *et al.* [90] claimed that multiple template-based homology modeling outweighs other methods for AT_1_R modeling.

Prokop *et al.* [91] proposed a new model of Ang II peptide binding to AT_1_R that correlates data from site directed mutagenesis and photolabeled experiments that were previously considered conflicting. Ang II binds AT_1_R and AT_2_R through a conserved initial binding mode involving amino acid Asn111 (consensus 325) of AT_1_R interacting with Tyr^4^ of Ang II and Lys199 and His256 (consensus 512 and 621) interacting with Phe^8^ of Ang II [91]. In both AT_1_R and AT_2_R, the Ang II peptide may internalize through Phe^8^ of Ang II propagating through the receptors’ conserved aromatic amino acids to the final photolabeled positioning relative to either AT_1_R at amino acid Asn294 (consensus 725) or AT_2_R at amino acid Leu138 (consensus 336). The authors conclude that understanding receptor activation provides valuable information for drug design and identification of other receptors that can potentially bind Ang II peptides [91].

A unique chemoselective photoaffinity labeling strategy, the methionine proximity assay (MPA), was used by Fillion *et al.* [92] to directly identify at physiological conditions a total of 38 discrete ligand/receptor contact residues that form the extracellular peptide-binding site of the activated AT_1_R. This experimental data set was used in homology modeling to guide the positioning of the Ang II peptide within several GPCR crystal structure templates. The CX-C chemokine receptor type 4 (CXCR4) accommodated the results better than the others templates evaluated; a *β*-hairpin fold in extracellular loop (ECL) 2, in conjunction with two extracellular disulfide bridges, appeared to open and shape the entrance of the ligand-binding site. The bound Ang II adopted a somewhat vertical binding mode allowing concomitant contacts across the extracellular surface and deep within the transmembrane domains (TMD) core of the receptor. The authors proposed that such a dualistic nature of GPCR interaction could be well suited for diffusible linear peptide ligands and a common feature of other peptidergic class A GPCRs [92]. Balakumar *et al.* [93] recently published a review article describing the structural and functional characteristics of Ang II and its analogues and antagonists and their interactions with amino residues in the AT_1_R.

Xie *et al.* [94] used the latest reported homologous chemokine receptors (PDB ID: 3ODU, 3OE0 and 3OE6) as templates to generate twenty models of AT_1_R receptor by multiple templates homology modeling. The docking results revealed that model 0020 possessed good affinities with these docked ARBs which are in accordance with both the IC_50_ inhibitor values and their curative effects. The results also showed more potent interactions between the model 0020 and its ARBs than those of ever reported results, such as hydrogen bonds, hydrophobic interactions, and especially cation-*π* interactions and *π*-*π* interactions. The authors, hypothesize that the structure of the model 0020 is quite close to its real crystal structure and the model 0020 may have the potential to be used for structure based drug design [94].

### 2.6. The Role of Membrane Bilayers in the Binding to AT Receptors

**Figure 13:**
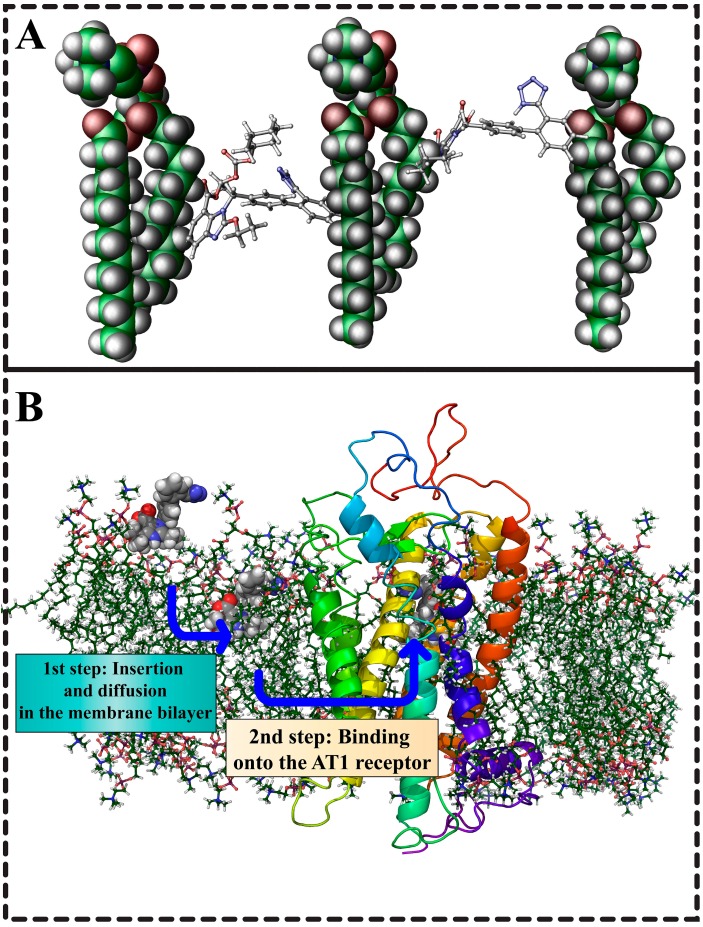
(**A**) candesartan cilexetil (left) and olmesartan (right) positioned in one leaflet of membrane bilayers. As it can be observed candesartan cilexetil is positioned deeper in membrane bilayers owing to its more hydrophobic nature. However, both molecules as they are amphipathic adopt polar and non-polar interactions. (**B**) Two step mechanism of action for ARBs. In the first step ARBs are postulated to embed themselves in the lipid matrix and in the second step are laterally diffused to the active site of AT_1_R.

AT_1_R antagonists act on the transmembrane region of the receptor site while Ang II interacts with both extracellular and intracellular site. The way that AT_1_R antagonists interact with their active site, however, remains uncharted. Do they directly approach the receptor or are they intercalated into the membrane core and then diffuse to the active site of the receptor (Figure 13B) How crucial is the membrane bilayer core for their action? Drug-membrane interactions along with drug-AT_1_R interactions are investigated to dissect the roles of these two components. The drug-membrane interactions show subtle but distinct differences between the thermal and dynamic properties of AT_1_R antagonists. The data suggest that each AT_1_R antagonist has a unique action on the membrane [95,96,97,98,99,100,101,102,103,104,105,106,107]. In the following figure, the interactions of candesartan cilexetil and olmesartan are illustrated as revealed from a combination of NMR spectroscopy, molecular modeling, Raman spectroscopy and Differential Scanning Calorimetry [108]. The study of the mechanism of losartan as it embeds in the lipid bilayers and approaches the AT_1_R has been recently studied [109]. Further work is needed to elaborate the issue of the preference of losartan into the two sites by using new models of AT_1_R as the one described by Matsoukas [89]. In our recent study, the application of 2D NOESY experiment led to the finding of the localization of AT_1_R antagonists (Figure 13A).

Molecular modeling enhances and promotes the field since it offers a better understanding of the interactions of drugs with AT_1_R and AT_2_R. The non-crystallized receptors demand the use of modeling to generate 3D low energy conformers of AT_1_R and AT_2_R. New approaches in the modeling of these receptors have recently been reported. Molecular Dynamics simulations are used to differentiate between the two step mechanism for drugs to reach the transmembrane active site of AT_1_R. The role of lipid bilayers in the AT_1_R antagonism is also studied using various biophysical methods.

## 3. Future Perspectives

The renin-angiotensin system is crucial for disease pathogenesis and cardiovascular control having as key players AT_1_R and AT_2_R. Recent studies provided evidence that these two receptors, besides their known implication in blood pressure regulation and hemodynamics, are actively participating in numerous other processes independent from hemodynamic effects. For instance the role of AT_1_R to promote aging has been recently described, suggesting potential benefits of AT_1_R antagonists to promote longevity [110]. Also AT_1_R antagonists are effective in reducing inflammation and autoimmunity in rheumatic diseases [111] and bear antitumor properties [15]. Concerning the function of the AT_2_R, this has been challenging owing to its low levels of expression and the limited availability of selective compounds targeting this receptor. Numerous literature evidence that the AT_2_R is strongly upregulated in cases of tissue damage such as myocardial infraction, brain ischemia, neuronal and vascular injury, highlights the high therapeutical potential of the AT_2_R in tissue protection and regeneration through regulating inflammation, fibrosis and apoptosis [56]. New results bring to light the beneficial effects of AT_2_R stimulation such as the protection of the myelin sheath in autoimmune central nervous system inflammation [112], inhibition of breast carcinoma cellular proliferation [26] and induction of analgesia [113]. Furthermore, not only the stimulation but also the inhibition of the AT_2_R is beneficial since very recently it was reported that the orally administered and highly selective AT_2_R antagonist EMA401 could be a novel treatment for postherpetic neuralgia [36].

These new findings highlight the need to develop novel and selective AT_1_R and AT_2_R analogues. Screening the recent literature for covering the design and synthesis of AT_1_R and AT_2_R ligands it has become evident that this field is not saturated and more effort is required to produce compounds bearing enhanced selectivity, potency and therapeutic efficacy. Still, there is no consensus of which model of the reported AT_1_R and AT_2_R has to be used by the medicinal chemists for the rational structure-based design and discovery of new selective drugs. It appears that the only solution to this problem will be the achievement of acquiring high resolution X-ray structures of these two receptors. Meanwhile, a significant effort must be made by the modelers to decide which model simulates more the biological one. The rationale used for the modelers is that docking and or molecular dynamics results should parallel the biological results. However, this has been claimed to exist for not only a unique model. This may reflect to the similarities of the proposed models.

Independently of the created models of the receptors, the synthetic chemists proceed with new ideas of synthesizing novel molecules. Indeed, these ideas were fruitful and bioactive molecules acting as AT_2_R agonists are already a reality. This effort to synthesize more selective drugs will certainly be continued. Another research activity which appears promising in the future is the synthesis of molecular hybrids and multitarget drugs. Due to the complexity of the systems that are involved in the cardiovascular diseases and others related to AT_1_R and AT_2_R the use of multitarget drugs will lead to beneficial aspects for treating these diseases avoiding in the same time side-effects. 
